# Hypocholesterolaemic effect of rat-administered oral doses of the isolated 7S globulins from cowpeas and adzuki beans

**DOI:** 10.1017/jns.2014.70

**Published:** 2015-02-16

**Authors:** Ederlan S. Ferreira, Ana Lucia S. Amaral, Aureluce Demonte, Cleslei F. Zanelli, Jessica Capraro, Marcello Duranti, Valdir A. Neves

**Affiliations:** 1 Department of Bromatological Analysis, School of Pharmacy, Federal University of Bahia, Barão de Jeremoabo Road, 147, 40170-115, Salvador, BA, Brazil; 2 Department of Food and Nutrition, São Paulo State University, Rodovia Araraquara – Jaú, km 1, 14801-902, Araraquara, SP, Brazil; 3 Department of Biological Sciences, School of Pharmaceutical Sciences, São Paulo State University, Rodovia Araraquara – Jaú, km 1, 14801-902, Araraquara, SP, Brazil; 4 Department of Food, Environmental and Nutritional Sciences (DeFENS), Università degli Studi di Milano, Via G. Celoria, 2, 20133, Milan, Italy

**Keywords:** *Vigna unguiculata*, *Vigna angularis*, 7S globulins, Cholesterol, Rats, 7S-A, adzuki bean 7S globulin, 7S-C, cowpea 7S globulin, HC, hypercholesterolaemic (high cholesterol and TAG), SREBP, sterol regulatory element-binding protein, STD, standard, TC, total cholesterol

## Abstract

The role of seed proteins, especially soyabean 7S globulins, in controlling dyslipidaemia is widely acknowledged. Amino acid sequence homology among the proteins of this family could reflect similar biological functions in other species. The aim of the present study was to unveil a hypolipidaemic effect of the 7S globulins from cowpeas (7S-C) and adzuki beans (7S-A), administered orally to rats fed a hypercholesterolaemic (HC; high cholesterol and TAG) diet for 28 d. A total of forty-five rats were divided into five groups (nine rats per group): (1) standard (STD) diet; (2) HC diet; (3) HC diet + 7S-C (300 mg/kg per d); (4) HC diet + 7S-A (300 mg/kg per d); and (5) HC diet + simvastatin (SVT; 50 mg/kg per d), as a control. Significant decreases in food intake and final body weight of rats receiving HC + 7S-C and HC + 7S-A diets compared with groups fed the HC and STD diets were observed. Significant decreases in serum total and non-HDL-cholesterol of 7S-C, 7S-A and SVT groups were also observed. HDL-cholesterol levels increased in the 7S-C, 7S-A and SVT groups, while hepatic cholesterol and TAG concentrations were significantly lower than in the HC diet group for the 7S-C-supplemented group only. Faecal excretions of fat and cholesterol in HC diet groups were considerably higher in animals consuming the 7S globulins. The results show that cowpea and adzuki bean 7S globulins promote cholesterol-decreasing effects in hypercholesterolaemic rats even at low dosages, as already observed for other legume seed storage proteins of this family. This main effect is discussed in relation to the possible mechanisms of action.

Dietary interventions have been used to control serum TAG and cholesterol, thus contributing to prevent CHD; in particular, diets containing soyabean and other legumes seeds have been associated with a reduction in the number of risk factors associated with these illnesses^(^^1^^–^^4^^)^. Soyabean proteins in particular have been studied, and these proteins have gained official recognition as potential reducers of risk factors for CVD in 1999, when the US Food and Drug Administration (FDA)^(^^5^^)^ accepted the recommendation on the intake of 25 g of soyabean protein per d for the prevention of these diseases. The FDA, based on a survey of clinical studies, indicated that daily consumption of these levels of soyabean protein may reduce total cholesterol (TC) and LDL-cholesterol^(^^6^^,^^7^^)^. However, proteins of other legumes have also shown some action on lipid metabolism and especially on cholesterol^(^^1^^,^^8^^,^^9^^)^, but most of these proteins have not been studied in great detail. The search of specific subfractions responsible for blood lipid-decreasing effects, as well as the mechanisms involved, is still the subject of many studies^(^^10^^–^^16^^)^. Once again, the dyslipidaemic-controlling effect of the soyabean β-conglycinin fraction, a 7S storage globulin, has been well documented both in animal models and human subjects^(^^3^^,^^10^^–^^12^^,^^17^^)^, but still no conclusive evidence on the mechanism/s involved has been put forward.

The 7S globulins of most leguminous seeds are composed of a subunit assortment yielding a molecular mass of approximately 150–220 kDa; the constituent subunits may vary in size and number within and among species^(^^18^^)^. Despite these differences, a considerable degree of sequence homology, when comparing partial and complete nucleotide sequences of genes and mRNA encoding for them, has been observed^(^^19^^)^. Various authors have described that the subunits of the 7S legume storage globulins have similar amino acid sequences^(^^20^^)^, and this may have led to the assumption that they can be cleaved in similar ways by digestive enzymes. Indeed, Nielsen *et al.*^(^^21^^)^ found that the cleavage sites of broad bean phaseolin by pepsin, trypsin and chymotrypsin are consistent with those of pea vicilin and soyabean β-conglycinin, all belonging to the 7S globulin family.

Previous studies have shown that β-conglycinin displays hypocholesterolaemic and hypolipidaemic action, when added to the diet as a unique source of protein or as a single daily administration^(^^10^^,^^12^^,^^22^^,^^23^^)^. More recently, various authors have demonstrated that the α' subunit is the component responsible for the activity^(^^10^^,^^24^^)^. The effects of soyabean 7S globulin were comparable with those observed with the drugs rosuvastatin, simvastatin and fibrates^(^^10^^,^^11^^,^^23^^)^. It is generally agreed that these effects on lipid metabolism may be due to peptides arising from the gastrointestinal digestion of protein. As a matter of fact, *in silico* simulated cleavage analyses showed that gastrointestinal digestion may lead to the production of peptides exhibiting similar biological activities as those detected with the soyabean 7S globulin (ES Ferreira, unpublished results). However, a unique region bearing the activity has not unequivocally been identified yet.

In the search of the biological activity of never-investigated species, we undertook the present study to monitor the effects of isolated 7S globulins from cowpeas and adzuki beans (7S-C and 7S-A, respectively), compared with simvastatin, on lipid parameters of rats fed a hyperlipidaemic and cholesterolaemic diet.

## Methods

### Flour preparation from cowpea and adzuki bean seeds

Cowpea (*Vigna unguiculata*, L) and adzuki bean (*Vigna angularis*, L) seeds were obtained from Empresa de Pesquisa Agropecuária de Minas Gerais at the Federal University of Viçosa, Minas Gerais, Brazil. The seeds were selected, soaked in distilled water (4°C/12 h), manually decorticated, dried at room temperature and powdered to 60 mesh sizes. The flours were stored to 4°C and used for protein extractions.

### Isolation of cowpea 7S globulin

The procedure consisted of the extraction of the cowpea flour with 1/20 (w/v) 0·1 m-NaCl in distilled water, adjusted to pH 7·5 with 2 m-NaOH. Stirring was applied for 1 h at room temperature. The suspension was then centrifuged at 10 000 ***g*** for 40 min at the same temperature. From this step, all following procedures were carried out at 4°C. The supernatant fraction was diluted 1/1 with distilled water; its pH was adjusted to 5·0 with 2 m-HCl and then kept overnight. A further centrifugation step as above allowed us to obtain a soluble fraction (albumins) and a pellet. The pellet was homogenised in water 1:20 (w (initial weight)/v) by using a Potter-Elvehjem homogeniser and stirred at 4°C for 10 min. Then, the pH was adjusted to 5·0 and the suspension centrifuged as above. The new pellet was dissolved in 0·1 m-NaCl 1:10 (w (initial weight)/v) at pH 7·0 and stirred at 4°C for 20 min. After further centrifugation as above, the pellet was discarded and the supernatant fraction was diluted 1/1 (v/v) with distilled water; the pH was adjusted to 5·0 and the solution was kept at 4°C overnight. The latter centrifugation step allowed us to recover a pellet, mainly consisting of 7S globulin, as will be shown. The pellet was dissolved in 0·2 m-NaCl at pH 7·0, and dialysed before freeze-drying.

### Isolation of adzuki bean 7S globulin

The adzuki bean flour was suspended in 1:20 (w/v) distilled water; the pH was adjusted to 7·5 and the suspension was stirred at room temperature for 30 min. The homogenate was then centrifuged as above. The supernatant fraction (S1) was kept and the pellet was solubilised in 1:10 (w (initial weight)/v) 0·5 m-NaCl, adjusted to pH 7·5 and stirred at room temperature for 30 min. Then the suspension was centrifuged as above and the supernatant fraction (S2) was mixed to S1. All subsequent procedures were performed at 4°C. The two mixed supernatant fractions were diluted 1/5 (v/v) with distilled water, homogenised as above, adjusted to pH 5 and kept at 4°C overnight. After centrifugation as above, the pellet was washed with distilled water and centrifuged once again. The new pellet was solubilised in 1:10 (w (initial weight)/v) 0·25 m-NaCl at pH 5·3 and stirred at 4°C for 20 min. After centrifugation as above, the soluble fraction was diluted 1/5 (v/v) with distilled water and homogenised as above; the pH was adjusted to 5·0 and the suspension was kept at 4°C overnight. After a final centrifugation as above, the supernatant fraction was discarded and the pellet was dissolved in 0·2 m-NaCl at pH 7·0, before dialysis and freeze-drying as above.

### SDS-PAGE

The homogeneity of the isolated proteins was performed on 10 % polyacrylamide gels containing 0·1 % SDS, in a Mini Protean II cell (Bio-Rad) and stained with Coomassie Brilliant Blue (R-250). Marker proteins of known molecular weight were: rabbit muscle phosphorylase B (94 kDa), bovine serum albumin (66 kDa), hen egg white albumin (45 kDa), bovine carbonic anhydrase (29 kDa), soyabean trypsin inhibitor (21·5 kDa) and hen egg white lysozyme (14·4 kDa).

### Diets and experimental protocol

All experiments were conducted in accordance with the Ethical Principles in Animal Research in the Guide for the Care and Use of Laboratory Animals^(^^25^^)^ and were approved by the Ethics Committee for Animal Research of the School of Pharmaceutical Sciences, São Paulo State University (UNESP) protocol 25/2009. A total of forty-five male Wistar rats aged 2 weeks (40–50 g body weight) were obtained from the Central Laboratory for Animals of the São Paulo State University (UNESP) at Botucatu (Brazil). The rats were fed a pelletised commercial (Purina^®^) diet for 2 weeks after arrival until reaching 150–160 g body weight. Afterwards, the animals were housed individually in stainless-steel cages in a room with a 12 h light–12 h dark cycle and a temperature of 23 ± 2°C. The rats were then divided into five groups (nine rats per group). A standard (STD) group was fed with a normal control diet, following the recommendations of the American Institute of Nutrition (AIN-93G) for growth^(^^26^^)^. Four hypercholesterolaemic (HC; high cholesterol and TAG) groups, namely HC-control, 7S-C, 7S-A and simvastatin, were fed with an AIN-93G diet, modified by adding 20 g/100 g coconut oil, 1 g/100 g cholesterol and 0·5 g/100 g cholic acid^(^^10^^,^^27^^)^ as described in Table 1. To three groups of HC animals, an oral dose of 300 mg/kg per d of the isolated 7S globulins and 50 mg/kg per d simvastatin were administered orally daily at 14·00 hours. The globulins and the drug were dissolved in saline buffer and the vehicle alone was given to one HC group. All animals were given food and water *ad libitum* during the experimental course (i.e. 28 d). The mentioned globulin doses were only slightly greater than those previously adopted with soyabean β-conglycinin^(^^10^^)^. Food consumption, weight gain, faecal excretion and feeding efficiency were measured each day of the trial. The feeding efficiency coefficient was calculated from the ratio of weight gain/daily intake × 100.

**Table 1.** Diet composition and treatments

		Hypercholesterolaemic diets			
	Standard diet*	HC diet*	HC diet + 7S-C	HC diet + 7S-A	HC diet + SVT
Ingredients (g/kg of chow)					
Casein†	200·0	200·0	200·0	200·0	200·0
Maize starch	514·5	384·5	384·5	384·5	384·5
Sucrose	100·0	100·0	100·0	100·0	100·0
Soyabean oil	70·0	–	–	–	–
Coconut oil	–	200·0	200·0	200·0	200·0
Cellulose powder	50·0	50·0	50·0	50·0	50·0
AIN-93G mineral mixture‡	35·0	35·0	35·0	35·0	35·0
AIN-93G vitamin mixture‡	10·0	10·0	10·0	10·0	10·0
l-Cystine	3·0	3·0	3·0	3·0	3·0
Choline bitartrate	2·50	2·50	2·50	2·50	2·50
Cholesterol†	10·0	10·0	10·0	10·0	10·0
Cholic acid†	5·0	5·0	5·0	5·0	5·0
Percentage energy					
Protein	22·56	19·40	19·88	19·88	19·40
Carbohydrate	61·63	41·78	41·53	41·53	41·78
Lipid	15·79	38·81	38·58	38·58	38·81
Energy (kJ/g)	16·69	19·41	19·50	19·50	19·41
Treatments (mg/kg per d)					
Cowpea β-vignin	–	–	300	–	–
Adzuki β-vignin	–	–	–	300	–
Simvastatin	–	–	–	–	50

HC diet, hypercholesterolaemic (high cholesterol and TAG) diet; HC diet + 7S-C, HC diet plus oral daily doses of cowpea 7S globulin (300 mg/kg per d); HC diet + 7S-A, HC diet plus oral daily doses of adzuki 7S globulin (300 mg/kg per d); HC diet + SVT, HC diet plus oral daily doses of simvastatin (50 mg/kg per d); AIN, American Institute of Nutrition.

* The standard diet was based on the recommendation of the AIN (AIN-93G) and the HC diet was bases on Nath's diet^(^^27^^)^ by adding 200 g/kg coconut oil, 10 g/kg cholesterol and 5 g/kg cholic acid.

† Sigma-Aldrich^®^, Co.

‡ PragSoluções^®^, Co.

### Blood and organ collection

On the last day, the animals were deprived of food for 12 h and euthanised by guillotine. Blood was then collected in tubes containing gel separator SST II (Vacutainer BD D^®^) and centrifuged at 1900 ***g*** for 15 min. The serum was separated, stored at –24°C and used for biochemical analysis. Epididymal adipose tissue, liver and heart were removed, washed immediately in cold saline buffer, dried and weighed, frozen and stored at –40°C for a period of less than 1 month for subsequent comparative analysis.

### Biochemical analyses of serum

Serum TC was measured by the liquid cholesterol CHOD-PAP (cholesterol oxidase-phenol + aminophenazone) method described by Stockbridge *et al.*^(^^28^^)^. Serum HDL-cholesterol was measured by the HDL-cholesterol precipitation method described by Assmann^(^^29^^)^. TAG were measured by the liquid TAG GPO-PAP (glycerol-3-phosphate oxidase-phenol + aminophenazone) method as described by Annoni *et al.*^(^^30^^)^. Serum glucose concentration was determined using a liquid glucose GOD-PAP (glucose oxidase-phenol + aminophenazone) method as described by Trinder^(^^31^^)^. All these colorimetric assays were carried out with commercially available kits (Laborlab^®^ Col). The non-HDL-cholesterol fraction (LDL-cholesterol + VLDL-cholesterol) was determined by difference between TC and HDL-cholesterol, and the atherogenic indexes (TC – HDL-cholesterol/HDL-cholesterol) were calculated as proposed by Liu *et al.*^(^^32^^)^. Hepato-somatic and visceral fat indexes were calculated by the following relationships, respectively: (liver weight/body weight) × 100, and (fat visceral weight/body weight) × 100, as described by Chen *et al.*^(^^33^^)^. Lipoprotein lipase activity was determined as described by Roe & Byler^(^^34^^)^ and glutamic pyruvic transaminase was determined as described by Tonks^(^^35^^)^ using a commercially available kit (Bioclin^®^ Co.). Serum insulin levels were analysed using a commercially available ultrasensitive rat insulin ELISA kit (DGR Instruments^®^ GmbH), as previously described previously by Korner *et al.*^(^^36^^)^. This assay had 100 % cross-reactivity to rat insulin.

### Analysis of hepatic and faecal lipids

Liver and faecal lipids were extracted with chloroform–methanol (2:1, v/v) according to the method previously described by Folch *et al.*^(^^37^^)^. TC and TAG were extracted by the method of Haug & Hostmark^(^^38^^)^. TC and TAG concentrations were measured as described earlier for serum analysis.

### Statistical analysis

All data are presented as mean values with their standard errors for nine values. The statistical analyses were performed using the SigmaStat^®^ 3.5 program (Dundas Software). Significant differences among the groups were determined by one-way ANOVA and Bonferroni *t* test multiple-range comparisons *v.* the HC group. A difference of *P* < 0·05 was considered statistically significant.

## Results

### Isolation of cowpea and adzuki bean 7S globulins for animal trials

**Fig. 1.**
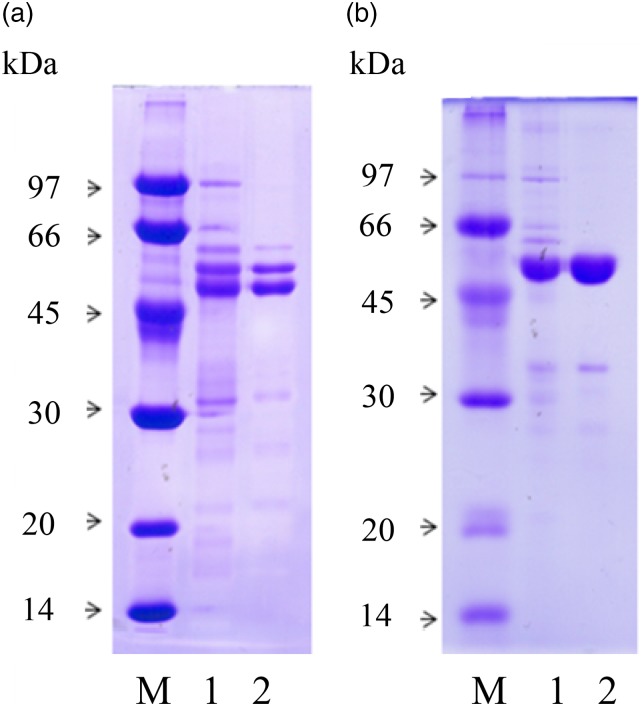
SDS-PAGE profile under reducing conditions of cowpea (a) and adzuki (b) 7S globulins. M, marker proteins; lanes 1, total protein extracts; lanes 2, isolated 7S proteins by the methods described. Loaded samples contained about 10 µg of the proteins.

In the present study, two novel isolation procedures of cowpea and adzuki bean main protein fractions, i.e. the 7S globulins, were used to generate sufficient amounts of proteins for animal trials (for details, see the Methods section). The results of SDS-PAGE conducted on the 7S globulins after separation from other protein components in cowpea and adzuki bean flours are shown in Fig. 1. The apparent molecular weights of the major polypeptides were about 55–56 kDa, and were consistent with the size of most polypeptide chains of the vicilin-like family^(^^19^^,^^21^^)^. With the two procedures approximately 8 and 3 g of 7S globulins from 100 g of cowpea and adzuki bean flours were obtained, respectively. The described procedures did not make use of any chromatographic step, which would have prevented large-scale protein preparations suitable for use in the present study. These preparations were judged sufficiently homogeneous and adequate for use in the *in vivo* experiments.

### Food intake, body weight and food efficiency

Effects of the daily administration of 7S-C, 7S-A and simvastatin on food consumption, weight gain, feeding efficiency ratio and faecal excretion in the animals of all groups, fed the diets for 28 d, are shown in Table 2. 7S-A and simvastatin produced a decrease of about 12 % on the final body weight of the animals, with a reduction on the average body-weight gain of 23 % with respect to the HC diet-fed animal group. 7S-C was effective too, though to a lesser extent. This fact could be attributed to the food intake reduction between groups in the period considered (Table 2). The HC group showed an increase of 72·8 and 72·2 % in the relative liver weight of rats and in the hepatosomatic index, respectively, relative to the STD diet-fed group (*P* < 0·001) (Table 2); on the other hand, only the 7S-A group presented a decrease of 18·6 % in the liver weight in relation to the HC group. No significant difference of the other parameters was found with respect to the HC group (Table 2).

**Table 2.** Body parameters measured in rats fed a hypercholesterolaemic (HC; high cholesterol and TAG) diet without and with oral daily doses of 7S globulins or simvastatin (SVT) for 4 weeks† (Mean values with their standard errors; nine rats per group)

	Standard diet		HC diet		HC diet + 7S-C		HC diet + 7S-A		HC diet + SVT	
	Mean	sem	Mean	sem	Mean	sem	Mean	sem	Mean	sem
Body weight (g/rat)										
Initial	184·30	3·73	183·80	3·87	182·10	3·90	182·00	3·23	182·10	3·62
Final	351·00	3·48	372·50	6·32	345·00*	7·34	328·50***	8·54	327·87***	6·90
Average gain	5·88**	0·08	6·79	0·23	5·85**	0·20	5·22***	0·20	5·26***	0·19
Food intake (g/rat per d)	18·92	0·31	18·19	0·47	16·42*	0·53	16·00**	0·46	15·66***	0·45
Faecal excretion (g/rat per d)	1·43	0·04	1·60	0·04	1·74	0·05	1·60	0·06	1·56	0·05
Feeding efficiency (%)	30·18***	0·67	36·00	0·74	36·93	0·76	34·74	0·86	34·85	0·86
Organ weights (g/rat)										
Liver	11·41***	0·32	19·72	0·72	19·34	0·65	16·06***	0·65	18·49	0·71
Epididymal adipose tissue	2·58***	0·21	5·16	0·33	4·16	0·33	4·31	0·34	4·72	0·31
Heart	1·21	0·04	1·23	0·03	1·19	0·04	1·11	0·05	1·06*	0·03
Tissue weight/body weight (%, g/100 g)										
Hepatic	3·20***	0·08	5·51	0·10	5·79	0·18	4·95*	0·12	5·43	0·18
Epididymal fat	0·71***	0·06	1·39	0·09	1·17	0·07	1·32	0·09	1·52	0·09
Cardiac	0·36	0·01	0·35	0·01	0·35	0·01	0·33	0·01	0·32	0·01

7S-C, cowpea 7S globulin; 7S-A, adzuki bean 7S globulin.

Mean value was significantly different from that of the HC diet group: * *P* < 0·05, ** *P* < 0·01, *** *P* < 0·001 (one-way ANOVA and Bonferroni *t* test multiple-range comparisons).

† For details of diets, see Table 1.

### Serum parameters

**Fig. 2.**
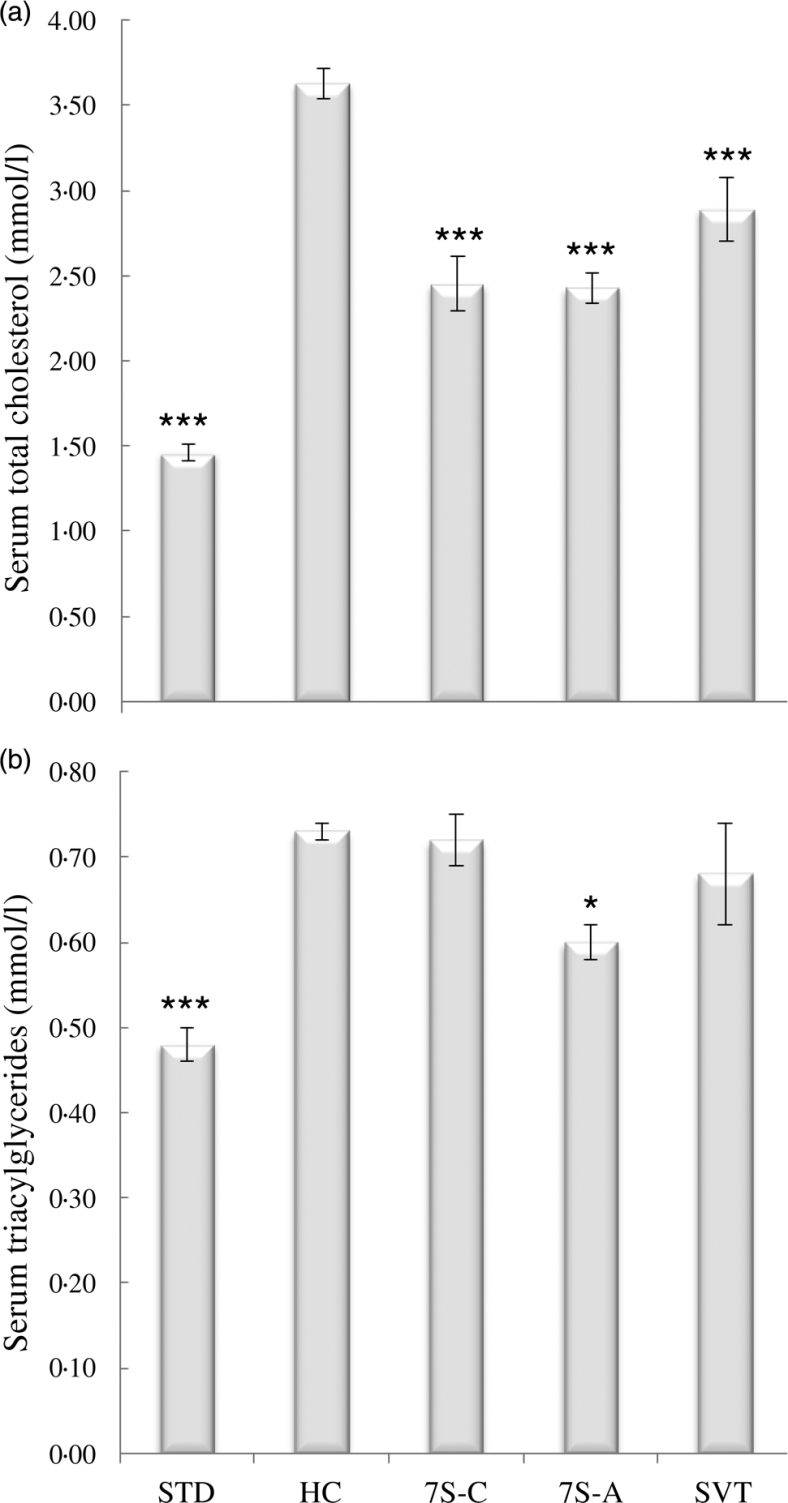
Serum total cholesterol (a) and TAG (b) levels of rats fed standard (STD) and hypercholesterolaemic (HC; high cholesterol and TAG) diets without (STD and HC) and with oral daily doses of cowpea 7S globulin (7S-C), adzuki 7S globulin (7S-A) or simvastatin (SVT) for 4 weeks. For details of diets, see Table 1. Values are means, with standard errors represented by vertical bars (nine rats per group). Mean value was significantly different from that of the HC diet group: * *P* < 0·05, *** *P* < 0·001 (one-way ANOVA and Bonferroni *t* test multiple-range comparisons).

In the rats fed the HC diet, serum lipid levels, both TC and TAG, were significantly higher than in those given the STD diet (*P* < 0·001) by the end of the 28 d treatment (Fig. 2). Conversely, the animals that were given the two globulins showed a reduction of 32·5 and 33 %, respectively (*P* < 0·001) in serum TC, while in those that received simvastatin the level was reduced by 20·3 % (*P* < 0·001). The levels of serum TAG were not significantly different in relation to the HC diet, except for the 7S-A group that showed a decrease of 17·8 % (*P* < 0·05).

Table 3 shows that the HC diet caused an increase of 5·86 times in the serum non-HDL-cholesterol of the animals, when compared with the STD diet (*P* < 0·001). Moreover, both 7S globulins reduced serum non-HDL-cholesterol by 46 % (*P* < 0·001), while only a 30·7 % reduction was observed with simvastatin (*P* < 0·001) relative to the HC diet. The HDL-cholesterol levels in the serum of the animals were affected by the HC diet that showed a decrease of 27 %, compared with the STD diet-fed group, while the 7S globulin-treated animals presented increases of 157 and 153 % for cowpeas and adzuki beans (*P* < 0·001), respectively, compared with the HC group, with values above the STD diet-fed group. By comparison, the animals from the simvastatin group increased these values only 18·5 % and below the values from the animals of the STD diet-fed group (Table 3). The atherogenic index, a marker of heart disease predisposition, increased 7·69 times by the effect of the hyperlipidic diet. Both proteins displayed a reducing effect on this parameter, with values 70·6 and 67 % lower than the HC group, respectively, and more efficiently than simvastatin treatment, as previously observed with soyabean proteins^(^^23^^,^^39^^)^.

**Table 3.** Serum, liver and faecal parameters and serum enzyme activities of rats fed a hypercholesterolaemic (HC; high cholesterol and TAG) diet without and with oral daily doses of 7S globulins or simvastatin (SVT) for 4 weeks† (Mean values with their standard errors; nine rats per group)

	Standard diet		HC diet		HC diet + 7S-C		HC diet + 7S-A		HC diet + SVT	
	Mean	sem	Mean	sem	Mean	sem	Mean	sem	Mean	sem
Serum (mmol/l)										
Non-HDL-cholesterol	0·50***	0·06	2·93	0·09	1·34***	0·16	1·37***	0·01	2·03***	0·25
HDL-cholesterol	0·96*	0·04	0·70	0·03	1·10***	0·05	1·07***	0·08	0·83	0·09
Atherogenic index‡	0·55***	0·08	4·23	0·26	1·24***	0·16	1·39***	0·20	2·89***	0·28
Glucose	6·27	0·15	5·86	0·15	6·27	0·12	5·42	0·27	6·85*	0·26
Insulin	0·60***	0·09	0·30	0·04	0·38	0·05	0·21	0·02	0·46	0·08
Liver (μmol/g)										
Total cholesterol	4·39***	0·20	23·37	0·91	20·18**	0·53	21·23	1·01	20·73*	0·38
TAG	45·82***	2·76	76·65	2·94	63·58***	2·24	74·54	1·64	72·10	1·53
Faecal (mg/d)										
Total fat	60·97***	1·26	152·99	1·97	170·99***	1·05	179·86***	0·73	168·96***	1·51
Total cholesterol	1·77***	0·03	21·93	0·09	23·55***	0·08	24·74***	0·10	23·62***	0·12
Enzyme activity (mmol/l per min)										
Lipoprotein lipase	5·42***	0·27	16·73	0·81	32·16***	2·68	27·22***	1·56	29·01***	1·31
Glutamate-pyruvate transaminase	12·28***	0·31	19·18	1·06	18·75	0·93	20·89	0·92	19·32	0·77

7S-C, cowpea 7S globulin; 7S-A, adzuki bean 7S globulin.

Mean value was significantly different from that of the HC diet group: * *P* < 0·05, ** *P* < 0·01, *** *P* < 0·001 (one-way ANOVA and Bonferroni *t* test multiple-range comparisons).

† For details of diets, see Table 1.

‡ Atherogenic index = log_10_ (TAG/HDL-cholesterol).

Serum glucose levels were not significantly affected in the trial, in spite of a decrease in the insulin levels of the groups fed the HC diet relative to the STD diet (*P* < 0·001) (Table 3). Table 3 shows also that lipoprotein lipase activities were significantly higher in all groups that received the HC diet; however, this effect was greater in the group treated with 7S-C. The groups that received the drug simvastatin or adzuki bean protein had a lower effect and were similar to each other. The activating effect of the globulin and simvastatin on the enzyme activity reached values close to twice that observed in the animals of the HC group.

### Liver parameters

The HC diet increased the levels of cholesterol and TAG in the liver relative to the STD diet (Table 3), while only the oral daily dose of 7S-C significantly reduced both parameters by 14 and 17 %, respectively (*P* < 0·05).

### Faecal excretion

Faecal excretions of fat and cholesterol were considerably higher in the groups consuming the HC diet (Table 3). Approximately 2·50 to 2·95 times more cholesterol was eliminated by the HC diet group compared with the STD diet-fed group. TAG excretion was 11·2 and 17·6 % greater with respect to the groups receiving the proteins of cowpea and adzuki beans, respectively; while the group that ingested the drug simvastatin was 10·4 % greater (*P* < 0·05). In the case of cholesterol, faecal excretion had a very large increase in the hypercholesterolaemic groups compared with the STD diet-fed group; anyway, the groups receiving the drug simvastatin or the proteins still showed greater excretion than the HC group (Table 3).

## Discussion

This study presents, as the main focus, comparative results on the cholesterol-lowering activities of two purified storage proteins from cowpeas and adzuki beans, respectively. Despite the limitations of the animal model and the very high-cholesterol diet used, the results are in line with similar ones on other legume seed proteins and highlight their potential in the cholesterolaemic control in humans, too. Indeed, while the use of drugs, such as statins, to positively affect cholesterol metabolism is well established and the mechanism of action is also well known^(^^40^^–^^42^^)^, for various reasons, including the lack of positive effect on HDL-cholesterol^(^^40^^)^, the need of alternative drugs and approaches is crucial. Among these, the interest for food components capable of effectively facing the cases of dyslipidaemia, sometimes in association with established drugs, in order to reduce their side effects, is growing^(^^41^^)^. The positive effects of soyabean proteins on the biochemical parameters related to altered metabolism of cholesterol and TAG are well described^(^^6^^,^^7^^,^^13^^,^^14^^,^^43^^–^^45^^)^; however, only in a few cases have these proteins, or other 7S and/or 11S globulin fractions, been administered to animals in a purified form and in small doses^(^^10^^–^^12^^,^^17^^,^^22^^,^^44^^,^^46^^)^. Moreover, despite interest in the use of β-conglycinin-homologous proteins from other legume seeds is great, only a few studies have been conducted on the hypocholesterolaemic effect of cowpea and adzuki bean proteins^(^^8^^,^^47^^–^^49^^)^. In an experiment using cowpeas as a unique protein source, the effect of the administration of crude flour and protein isolate resulted in a significant decrease in serum TC and non-HDL-cholesterol and an increase of faecal cholesterol in hamsters^(^^8^^)^. Contrary to this, Olivera *et al.*^(^^48^^)^ verified that a protein isolate from cowpeas, as the single protein source, did not alter the levels of TC and TAG in the serum of rats. In this case, however, animals fed a normolipidaemic diet were used. Meanwhile, Mahadevappa & Raina^(^^47^^)^ observed that the addition of whole cowpea flour, as a source of protein, in a hypercholesterolaemic and hyperlipidaemic diet caused a reduction of up to 55 % in serum TC in rats. Concerning the other source of protein, namely adzuki beans, Chau *et al.*^(^^49^^)^ observed no changes in serum TC, LDL-cholesterol and HDL-cholesterol levels in hamsters fed a hypercholesterolaemic diet by using a protein concentrate of this seed.

In order to clarify these controversial findings, in the present study only extensively purified 7S-C and 7S-A were given as a single daily oral dose to rats submitted to a hypercholesterolaemic and hyperlipidaemic diet for 28 d. It is worth remarking here that the administered doses represented only 2·75 % of the total protein ingested daily by the animals.

A number of body and serum parameters were evaluated in the present study. Among them, a significant decrease in food intake in rats receiving 7S-C and 7S-A compared with HC and STD diet-fed groups was observed. The lower daily food intake is probably responsible for the reduction in the average weight gain and final weight of the treated rats. Interestingly, some studies^(^^50^^)^ found that soyabean β-conglycinin presented a suppressing effect on food intake in rats via an increased cholecystokinin secretion. Hira *et al.*^(^^51^^)^ verified that the 3 g intake of hydrolysed β-conglycinin was effective to increase satiety and reduce feelings of hunger in healthy human subjects. Also, Kohno *et al.*^(^^17^^)^ in a study with 126 volunteers found that β-conglycinin consumption for 4 weeks, in the form of a sweet, resulted in a significant reduction of visceral fat, thus contributing to the prevention of obesity. Additionally, β-conglycinin-enriched soyabean seeds can provide hydrolysates that limit fat accumulation in fat cells and inflammatory pathways *in vitro*, and therefore warrant further studies as a healthful food^(^^52^^)^.

A key point in the present study is the finding that isolated 7S-C and 7S-A are capable of reducing serum cholesterol in hypercholesterolaemic rats as efficiently as simvastatin, as previously observed with rosuvastatin and β-conglycinin^(^^23^^)^. Remarkably, these findings were obtained with a much lower dosage than that used in studies where β-conglycinin was used as the only source of protein in the diet^(^^6^^,^^13^^,^^14^^,^^44^^)^. Similarly, Duranti *et al.*^(^^10^^)^ verified that low dosages of β-conglycinin and its α' subunit administered to hypercholesterolaemic rats decreased by 50 % the serum levels of cholesterol and TAG, comparably to clofibrate. Conversely, the reduction in TAG levels was greater for both β-conglycinin and 7S-A, while 7S-C and simvastatin did not significantly affect this parameter. These differences may denote subtle but relevant differences in the mechanism of action of the different globulins.

The increased faecal excretion of TC observed in the groups receiving the two globulins and simvastatin, compared with the HC group, is in line with the reduction of serum cholesterol levels. A similar conclusion has already been put forward with β-conglycinin^(^^14^^)^, glycinin^(^^44^^)^, black soya peptides^(^^53^^)^ and other legumes^(^^54^^)^. Since a similar cholesterol-lowering effect has been monitored with both soyabean protein hydrolysate and the intact corresponding protein^(^^55^^)^, bioactive peptides may affect both cholesterol metabolism and increase in faecal excretion, similarly to dietary fibres.

Increased levels of lipoprotein lipase observed in the animals that received 7S-C and 7S-A and simvastatin may be related to the reduction of TC and TAG in the serum of the animals, as also noted by Mochizuki *et al.*^(^^56^^)^.

7S-C and 7S-A promoted the reduction of the non-HDL-cholesterol fraction, 40 % greater than with β-conglycinin^(^^23^^)^, 60 % greater than with the two statins and close to that of fenofibrate^(^^11^^)^, in the same experimental model.

The reduction in serum insulin levels observed in the animals of the HC diet-fed group (Table 3) could have favoured the high values of the lipoproteins (non-HDL and HDL) in the serum. Various authors have observed the LDL-reducing effect of β-conglycinin, *in vivo* and *in vitro*. The possible mechanism has been inferred from the findings that the protein/peptides lead to an increase in mRNA expression of LDL receptors^(^^7^^,^^16^^,^^22^^,^^45^^,^^56^^,^^57^^)^, thus causing a more efficient removal of the particles from the bloodstream, promoting the secretion of apoB-100 and consequently decreasing the secretion of VLDL-cholesterol^(^^56^^)^.

The HDL-cholesterol fraction plays the important function of delivering cholesterol to the liver, thus increasing its catabolism. Statins, regardless of the dose, have been shown to have a small influence on the levels of the HDL-cholesterol fraction^(^^58^^)^. Conversely, the combination statin/β-conglycinin showed a positive effect, resulting in a higher level of HDL-cholesterol in the serum of rats than the drug alone^(^^23^^)^, and suggesting a synergic effect of the two molecules on cholesterol metabolism. The most surprising effect observed with 7S-C and 7S-A was with this parameter: indeed they increased HDL-cholesterol by 56 and 53 %, respectively, while simvastatin showed an expected minor effect (Table 3). Similarly, these proteins were more efficient than simvastatin in reducing the atherogenic index, as also observed with soyabean 7S and 11S isolated proteins^(^^23^^,^^39^^)^. This finding would suggest a synergic effect of these globulins with statins, as observed for β-conglycinin. Interestingly, the HDL-cholesterol values obtained with the globulins overcome even those of the group fed the normolipidaemic diet.

Despite the many studies with isolated 7S soyabean globulins and other seed proteins, a unique mechanism of action cannot be envisaged yet. As mentioned, different authors have shown a biochemical action via the inhibition of certain enzymes in the metabolism of cholesterol and TAG^(^^2^^,^^13^^,^^14^^,^^42^^)^. Also an action on gene expression, specially on hepatic mRNA of some key enzymes of sterol metabolism and LDL receptors^(^^22^^,^^24^^,^^44^^,^^52^^,^^56^^,^^57^^)^, as well as through modulation of transcription factors, such as sterol regulatory element-binding protein (SREBP)-1c and SREBP-2^(^^2^^,^^52^^,^^56^^)^, have been described. The observed hypocholesterolaemic effects of 7S-C and 7S-A matched those of β-conglycinin, both *in vivo* and *in vitro*^(^^10^^–^^12^^,^^17^^,^^22^^,^^23^^,^^44^^,^^52^^,^^56^^,^^57^^)^. Still, hypothesising a mechanism of action is untimely. Nevertheless, globulin administration in an oral daily dose suggests that biologically active peptides, arising from their digestion, could be responsible for the observed effects, as already mentioned by several authors^(^^1^^,^^16^^,^^22^^,^^42^^,^^55^^,^^56^^)^. As a matter of facts, preliminary results in our laboratory would support the conclusion that these proteins affect mRNA levels of various enzymes such as fatty acid synthase, 3-hydroxy-3-methyl-glutaryl-CoA (HMG-CoA) reductase, HMG-CoA synthetase, as well as regulatory factors including SREBP-1c, SREBP-2 and LDL receptors (ES Ferreira, unpublished results). On the other hand, the globulin-induced increased faecal cholesterol and TAG excretion associated with lower cholesterol concentrations in the liver, especially for 7S-C, speaks in favour of a decreased re-absorption of intestinal lipids, as already described for soyabean bioactive peptides^(^^59^^,^^60^^)^, and also focuses on possibly different mechanisms of action between the two proteins, which will require further experimental work. In this respect, the interaction of extracted seed globulins with minor amounts of non-protein components, such as fibres, phytates and saponins, cannot be excluded, though the purity of 7S-C and 7S-A was 94 and 96 %, respectively (not shown).

Although the mechanisms of the cholesterol-lowering effects of legume seed storage globulins have not been made clear yet, it can be argued that, due to the large size of these molecules and their susceptibility to proteolytic enzymes, the observed effects could be attributed to peptides derived from their gastrointestinal digestion. Once absorbed and transported to the liver, these peptides could modulate the homeostasis of cholesterol, as already discussed by other authors for some proteins^(^^60^^,^^61^^)^. Alternatively, other studies concluded that the reduction of serum cholesterol might be a consequence of a direct interaction between these peptides and cholesterol, or its catabolites, thus promoting its excretion^(^^62^^,^^63^^)^. Further studies are needed to unequivocally identify the mechanism of action of these proteins on lipid metabolism.

In conclusion, the present study first showed that 7S-C and 7S-A in an isolated form and at low dosages are effective in reducing serum cholesterol levels in hypercholesterolaemic rats, thus confirming that this class of proteins, regardless of the species, may exert similar biological activities in animal models.
